# Sanatoria and Convalescent Homes

**Published:** 1914-03-14

**Authors:** Edith Mawe

**Affiliations:** Hon. Lady Superintendent. Royal West of England Sanatorium, Weston-super-Mare


### THE EDITOR'S LETTER-BOX.

### Sanatoria and Convalescent Homes.

*To the Editor of* The Hospital.

Sir,—Will you kindly allow me to correct the wrong impression given by the paragraph in your issue of February 28, headed "Sanatoria and Convalescent Homes"? I quite agree that tuberculosis should be treated in separate institutions, and as far back as 1909 my committee decided to reject cases of defined phthisis pulmonalis, though still accepting for treatment cases of threatened phthisis, these being certified as free from phthisis.—Yours faithfully,

Edith Mawe,
Hon. Lady Superintendent.

Royal West of England Sanatorium,
Weston-super-Mare, March 4, 1914.

[The statement in the paragraph was: "Fewer pulmonary cases are being received in consequence of the number of new sanatoria now opened, while the number of surgical cases tends to increase. But, then, this institution, we understand, has never limited itself entirely to the various forms of tuberculosis. Since it is desirable that the latter disease should be treated in special institutions, the tendency here noticed is in the right direction. There should be no ground for confusion between sanatoria proper and general convalescent homes." Surely no "wrong impression" can be created by the mention of these facts.—Ed. The Hospital.]

